# Large language models could change the future of behavioral healthcare: a proposal for responsible development and evaluation

**DOI:** 10.1038/s44184-024-00056-z

**Published:** 2024-04-02

**Authors:** Elizabeth C. Stade, Shannon Wiltsey Stirman, Lyle H. Ungar, Cody L. Boland, H. Andrew Schwartz, David B. Yaden, João Sedoc, Robert J. DeRubeis, Robb Willer, Johannes C. Eichstaedt

**Affiliations:** 1 Dissemination and Training Division, National Center for PTSD, VA Palo Alto Health Care System, Palo Alto, CA, USA; 2 Department of Psychiatry and Behavioral Sciences, Stanford University, Stanford, CA, USA; 3 Institute for Human-Centered Artificial Intelligence & Department of Psychology, Stanford University, Stanford, CA, USA; 4 Department of Computer and Information Science, University of Pennsylvania, Philadelphia, PA, USA; 5 Department of Computer Science, Stony Brook University, Stony Brook, NY, USA; 6 Department of Psychiatry and Behavioral Sciences, Johns Hopkins University School of Medicine, Baltimore, MD, USA; 7 Department of Technology, Operations, and Statistics, New York University, New York, NY, USA; 8 Department of Psychology, University of Pennsylvania, Philadelphia, PA, USA; 9 Department of Sociology, Stanford University, Stanford, CA, USA

**Keywords:** Psychology, Psychiatric disorders, Psychology, Technology

## Abstract

Large language models (LLMs) such as Open AI’s GPT-4 (which power ChatGPT) and Google’s Gemini, built on artificial intelligence, hold immense potential to support, augment, or even eventually automate psychotherapy. Enthusiasm about such applications is mounting in the field as well as industry. These developments promise to address insufficient mental healthcare system capacity and scale individual access to personalized treatments. However, clinical psychology is an uncommonly high stakes application domain for AI systems, as responsible and evidence-based therapy requires nuanced expertise. This paper provides a roadmap for the ambitious yet responsible application of clinical LLMs in psychotherapy. First, a technical overview of clinical LLMs is presented. Second, the stages of integration of LLMs into psychotherapy are discussed while highlighting parallels to the development of autonomous vehicle technology. Third, potential applications of LLMs in clinical care, training, and research are discussed, highlighting areas of risk given the complex nature of psychotherapy. Fourth, recommendations for the responsible development and evaluation of clinical LLMs are provided, which include centering clinical science, involving robust interdisciplinary collaboration, and attending to issues like assessment, risk detection, transparency, and bias. Lastly, a vision is outlined for how LLMs might enable a new generation of studies of evidence-based interventions at scale, and how these studies may challenge assumptions about psychotherapy.

## Introduction

Large language models (LLMs), built on artificial intelligence (AI) – such as Open AI’s GPT-4 (which power ChatGPT) and Google’s Gemini – are breakthrough technologies that can read, summarize, and generate text. LLMs have a wide range of abilities, including serving as conversational agents (chatbots), generating essays and stories, translating between languages, writing code, and diagnosing illness^1^. With these capacities, LLMs are influencing many fields, including education, media, software engineering, art, and medicine. They have started to be applied in the realm of behavioral healthcare, and consumers are already attempting to use LLMs for quasi-therapeutic purposes^2^.

Applications incorporating older forms of AI, including natural language processing (NLP) technology, have existed for decades^3^. For example, machine learning and NLP have been used to detect suicide risk^4^, identify the assignment of homework in psychotherapy sessions^5^, and identify patient emotions within psychotherapy^6^. Current applications of LLMs in the behavioral health field are far more nascent – they include tailoring an LLM to help peer counselors increase their expressions of empathy, which has been deployed with clients both in academic and commercial settings^2,7^. As another example, LLM applications have been used to identify therapists’ and clients’ behaviors in a motivational interviewing framework^8,9^.

Similarly, while algorithmic intelligence with NLP has been deployed in patient-facing behavioral health contexts, LLMs have not yet been heavily employed in these domains. For example, mental health chatbots Woebot and Tessa, which target depression and eating pathology respectively^10,11^, are rule-based and do not use LLMs (i.e., the application’s content is human-generated, and the chatbot’s responds based on predefined rules or decision trees^12^). However, these and other existing chatbots frequently struggle to understand and respond to unanticipated user responses^10,13^, which likely contributes to their low engagement and high dropout rates^14,15^. LLMs may hold promise to fill some of these gaps, given their ability to flexibly generate human-like and context-dependent responses. A small number of patient-facing applications incorporating LLMs have been tested, including a research-based application to generate dialog for therapeutic counseling^16,17^, and an industry-based mental-health chatbot, Youper, which uses a mix of rule-based and generative AI^18^.

These early applications demonstrate the potential of LLMs in psychotherapy – as their use becomes more widespread, they will change many aspects of psychotherapy care delivery. However, despite the promise they may hold for this purpose, caution is warranted given the complex nature of psychopathology and psychotherapy. Psychotherapy delivery is an unusually complex, high-stakes domain vis-à-vis other LLM use cases. For example, in the productivity realm, with a “LLM co-pilot” summarizing meeting notes, the stakes are failing to maximize efficiency or helpfulness; in behavioral healthcare, the stakes may include improperly handling the risk of suicide or homicide.

While there are other applications of artificial intelligence that may involve high-stakes or life-or death decisions (e.g., self-driving cars), prediction and mitigation of risk in the case of psychotherapy is very nuanced, involving complex case conceptualization, the consideration of social and cultural contexts, and addressing unpredictable human behavior. Poor outcomes or ethical transgressions from clinical LLMs could run the risk of harming individuals, which may also be disproportionately publicized (as has occurred with other AI failures^19^), which may damage public trust in the field of behavioral healthcare.

Therefore, developers of clinical LLMs need to act with special caution to prevent such consequences. Developing responsible clinical LLMs will be a challenging coordination problem, primarily because the technological developers who are typically responsible for product design and development lack clinical sensitivity and experience. Thus, behavioral health experts will need to play a critical role in guiding development and speaking to the potential limitations, ethical considerations, and risks of these applications.

Presented below is a discussion on the future of LLMs in behavioral healthcare from the perspective of both behavioral health providers and technologists. A brief overview of the technology underlying clinical LLMs is provided for the purposes of both educating clinical providers and to set the stage for further discussion regarding recommendations for development. The discussion then outlines various applications of LLMs to psychotherapy and provides a proposal for the cautious, phased development and evaluation of LLM-based applications for psychotherapy.

## Overview of clinical LLMs

Clinical LLMs could take a wide variety of forms, spanning everything from brief interventions or circumscribed tools to augment therapy, to chatbots designed to provide psychotherapy in an autonomous manner. These applications could be patient-facing (e.g., providing psychoeducation to the patient), therapist-facing (e.g., offering options for interventions from which the therapist could select), trainee-facing (e.g., offering feedback on qualities of the trainee’s performance), or supervisor/consultant facing (e.g., summarizing supervisees’ therapy sessions in a high-level manner).

### How language models work

Language models, or computational models of the probability of sequences of words, have existed for quite some time. The mathematical formulations date back to^20^ and original use cases focused on compressing communication^21^ and speech recognition^22–24^. Language modeling became a mainstay for choosing among candidate phrases in speech recognition and automatic translation systems but until recently, using such models for *generating* natural language found little success beyond abstract poetry^24^.

### Large language models

**Fig. 1:**
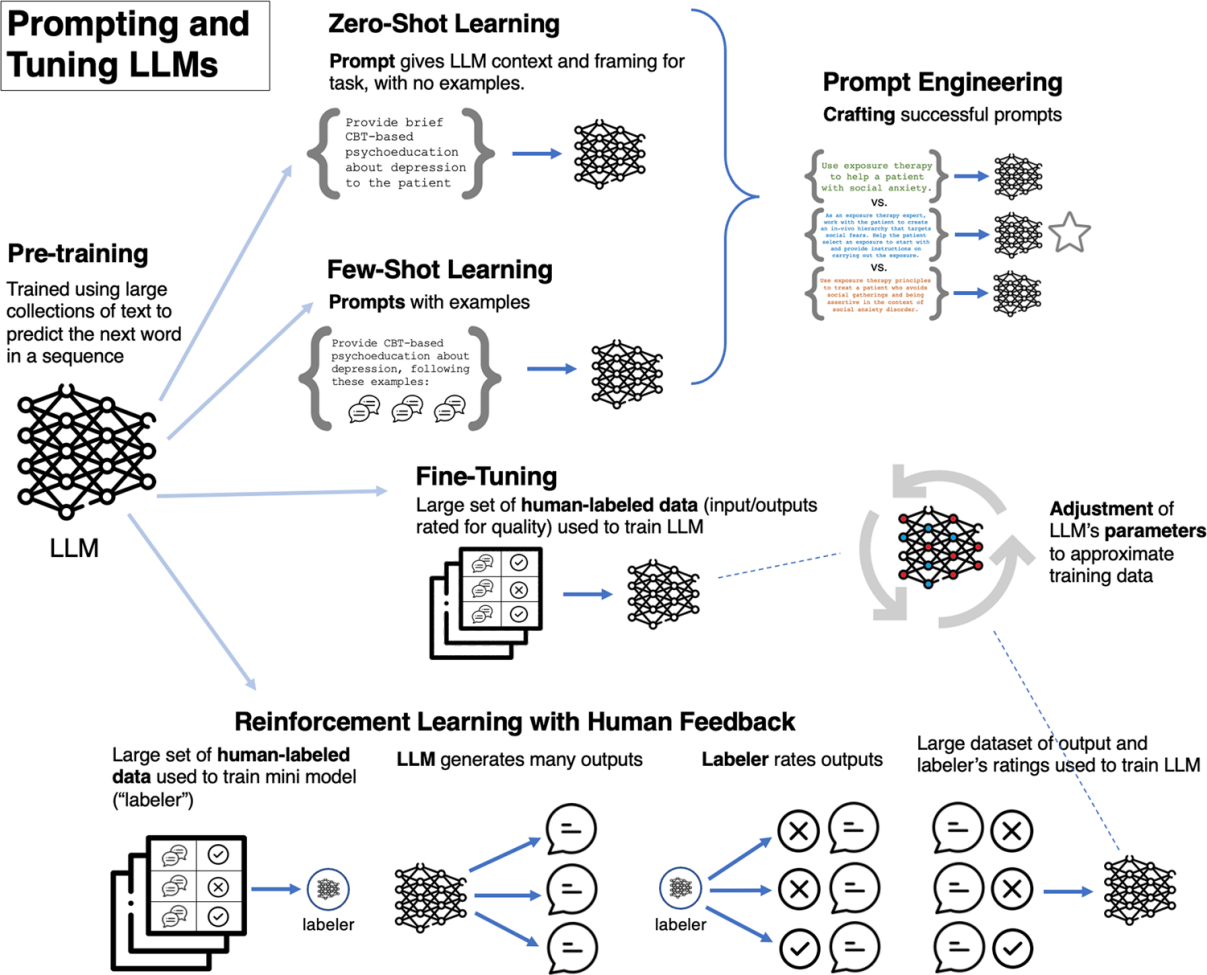
Methods for tailoring clinical large language models. Figure was designed using image components from Flaticon.com.

The advent of *large* language models, enabled by a combination of the deep learning technique transformers^25^ and increases in computing power, has opened new possibilities^26^. These models are first trained on massive amounts of data^27,28^ using “unsupervised” learning in which the model’s task is to predict a given word in a sequence of words. The models can then be tailored to a specific task using methods, including prompting with examples or fine-tuning, some of which use no or small amounts of task-specific data (see Fig. 1)^28,29^. LLMs hold promise for clinical applications because they can parse human language and generate human-like responses, classify/score (i.e., annotate) text, and flexibly adopt conversational styles representative of different theoretical orientations.

### LLMs and psychotherapy skills

**Fig. 2:**
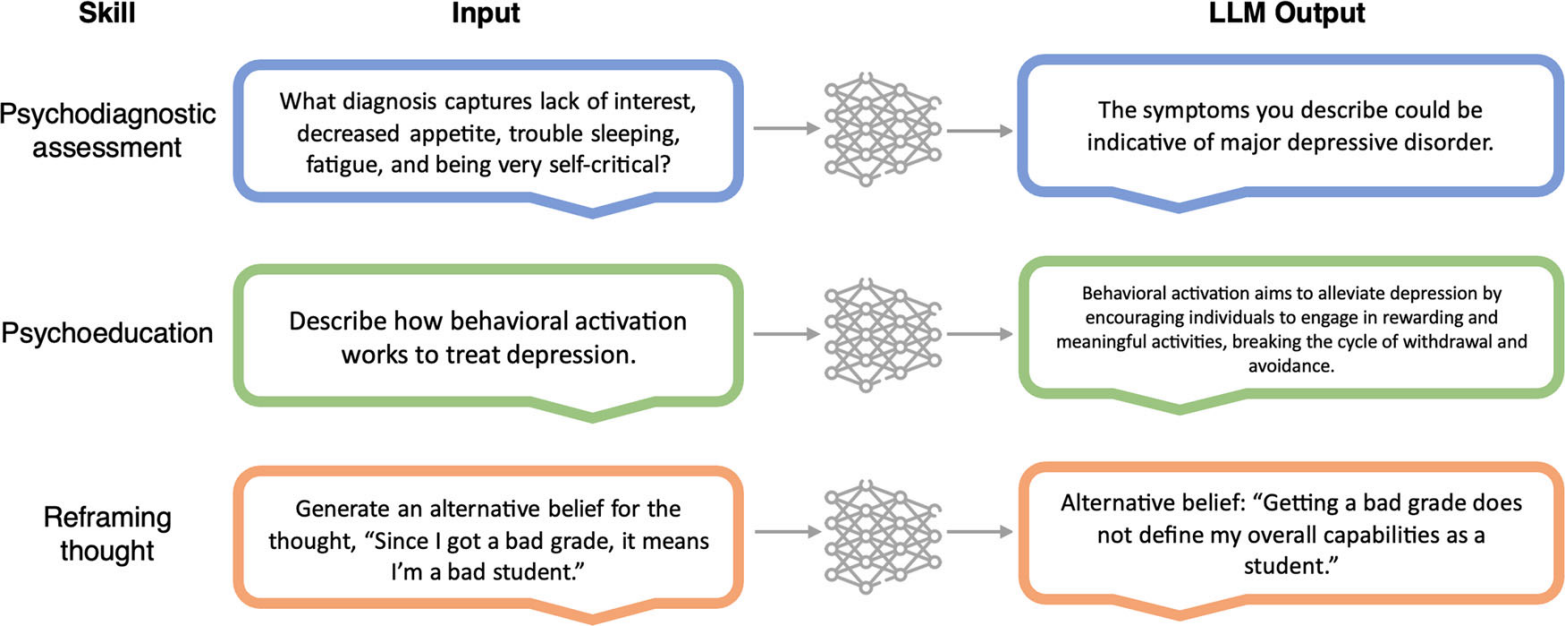
Example clinical skills of large language models. *Note*. Figure was designed using image component from Flaticon.com.

For certain use cases, LLM show a promising ability to conduct tasks or skills needed for psychotherapy, such as conducting assessment, providing psychoeducation, or demonstrating interventions (see Fig. 2). Yet to date, clinical LLM products and prototypes have not demonstrated anywhere near the level of sophistication required to take the place of psychotherapy. For example, while an LLM can generate an alternative belief in the style of CBT, it remains to be seen whether it can engage in the type of turn-based, Socratic questioning that would be expected to produce cognitive change. This more generally highlights the gap that likely exists between simulating therapy skills and implementing them effectively to alleviate patient suffering. Given that psychotherapy transcripts are likely poorly represented in the training data for LLMs, and that privacy and ethical concerns make such representation challenging, prompt engineering may ultimately be the most appropriate fine-tuning approach for shaping LLM behavior in this manner.

## Clinical LLMs: stages of integration

**Fig. 3:**
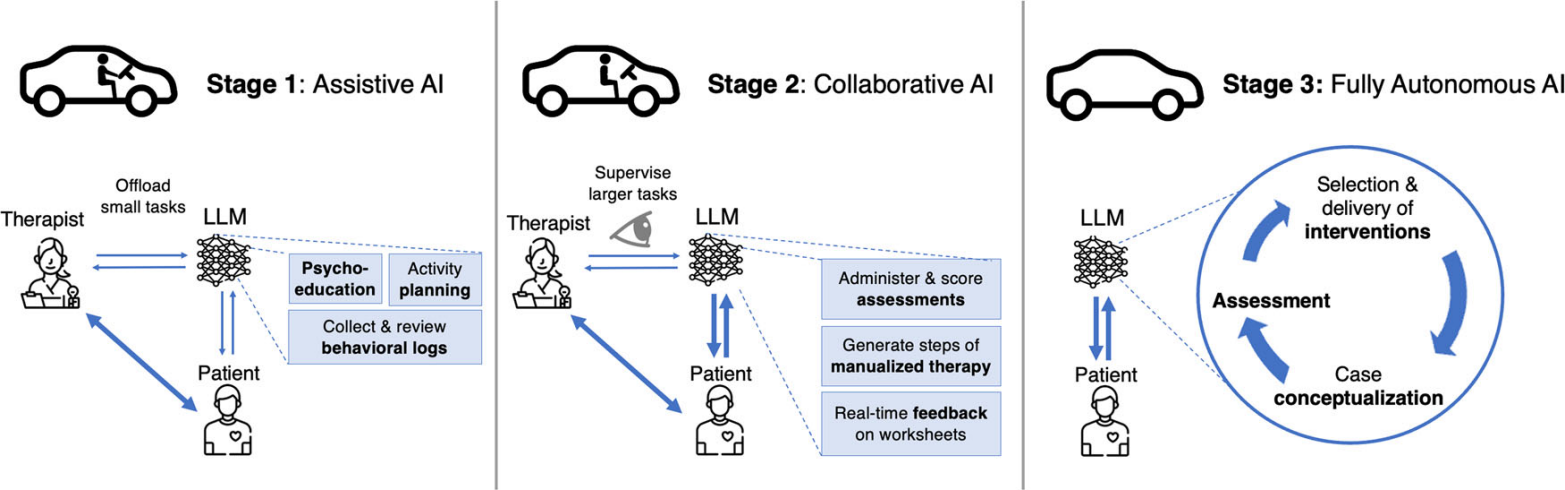
Stages of integrating large language models into psychotherapy. Figure was designed using image components from Flaticon.com.

The integration of LLMs into psychotherapy could be articulated as occurring along a continuum of stages spanning from assistive AI to fully autonomous AI (see Fig. 3 and Table 1). This continuum can be illustrated by models of AI integration in other fields, such as those used in the autonomous vehicle industry. For example, at one end of this continuum is the assistive AI (“machine in the loop”) stage, wherein the vehicle system has no ability to complete the primary tasks – acceleration, braking, and steering – on its own, but provides momentary assistance (e.g., automatic emergency breaking, lane departure warning) to increase driving quality or decrease burden on the driver. In the collaborative AI (“human in the loop”) stage, the vehicle system aids in the primary tasks, but requires human oversight (e.g., adaptive cruise control, lane keeping assistance). Finally, in fully autonomous AI, vehicles are self-driving and do not require human oversight. The stages of LLM integration into psychotherapy and their related functionalities are described below.

**Table 1:** Stages of Development of Clinical LLMs

Stage	Car Analogy	Characteristics of Assessment	Intervention Focus/Scope	Intervention Nature	Clinical Example	Potential Risks or Costs
Assistive AI (*“machine in the loop”*)	AI-based features (e.g., automatic emergency breaking, lane departure warning) in the vehicle.	Standalone, modularized (e.g., assessments hand-picked by therapist and administered by survey).	Limited to concrete/ circumscribed (e.g., activity planning).	No full intervention packages; limited to components of interventions.	LLM trained to conduct skills from CBT-I might converse with the patient to collect their sleep diary data from the previous week to expedite a traditional therapy session.	Overhead and complexity for therapist for AI supervision.
Collaborative AI (*“human in the loop”*)	Vehicle mostly completing the primary task; human in the driver seat actively monitors the vehicle’s progress and overrides it as needed (e.g., adaptive cruise control, lane keeping assist).	Increasingly integrated (e.g., assessments recommended by LLM and summarized with context for therapist review).	Includes less concrete, more abstract interventions (e.g., planning and processing exposures).	Limited to structured/standardized (e.g., CBT for insomnia).	CBT-I LLM might generate a) an overview of the sleep diary data, b) a rationale for sleep restriction and stimulus control, and c) a sleep schedule prescription based on the diary data. This content would be reviewed and tailored by the psychotherapist before being discussed with the patient.	Drafts that require significant corrections may not save much time; Busy therapists may fail to check or tailor content, especially if given higher caseloads due to AI assistance.
Fully autonomous AI	Fully autonomous vehicles that operate without direct human oversight.	Fully integrated, informs intervention (e.g., unobtrusive, automated symptom assessment running in background).	Includes very abstract/diffuse interventions (e.g., Socratic questioning).	Includes unstructured/unstandardized (e.g., acceptance and commitment therapy, idiographic or modular approaches).	LLM could implement a full course of CBT-I. The LLM would directly deliver multi-session therapy interventions and content to the patient, which would not be subject to tailoring or initial oversight by the psychotherapist.	Critical information could be missed (e.g., suicide risk); Provision of inappropriate or harmful care.

*AI* artificial intelligence, *LLM* large language model, *CBT-I* cognitive behavioral therapy for insomnia.

### Stage 1: assistive LLMs

At the first stage in LLM integration, AI will be used as a tool to assist clinical providers and researchers with tasks that can easily be “offloaded” to AI assistants (Table 1; first row). As this is a preliminary step in integration, relevant tasks will be low-level, concrete, and circumscribed, such that they present a low level of risk. Examples of tasks could include assisting with collecting information for patient intakes or assessment, providing basic psychoeducation to patients, suggesting text edits for providers engaging in text-based care, and summarizing patient worksheets. Administratively, systems at this stage could also assist with clinical documentation by drafting session notes.

### Stage 2: collaborative LLMs

Further along the continuum, AI systems will take the lead by providing or suggesting options for treatment planning and much of the therapy content, which humans will use their professional judgement to select from or tailor. For example, in the context of a text- or instant-message delivered structured psychotherapeutic intervention, the LLM might generate messages containing session content and assignments, which the therapist would review and adapt as needed before sending (Table 1; second row). A more advanced use of AI within the collaborative stage may entail a LLM providing a structured intervention in a semi-independent manner (e.g., as a chatbot), with a provider monitoring the discussion and stepping in to take control of the conversation as needed. The collaborative LLM stage has parallels to “guided self-help” approaches^30^.

### Stage 3: fully autonomous LLMs

In the fully autonomous stage, AIs will achieve the greatest degree of scope and autonomy wherein a clinical LLM would perform a full range of clinical skills and interventions in an integrated manner without direct provider oversight (Table 1; third row). For example, an application at this stage might theoretically conduct a comprehensive assessment, select an appropriate intervention, and deliver a full course of therapy with no human intervention. In addition to clinical content, applications in this stage could integrate with the electronic health record to complete clinical documentation and report writing, schedule appointments and process billing. Fully autonomous applications offer the most scalable treatment method^30^.

### Progression across the stages

Progression across the stages may not be linear; human oversight will be required to ensure that applications at greater stages of integration are safe for real world deployment. As different forms of psychopathology and their accompanying interventions vary in complexity, certain types of interventions will be simpler than others to develop as LLM applications. Interventions that are more concrete and standardized may be easier for models to deliver (and may be available sooner), such as circumscribed behavior change interventions (e.g., activity scheduling), as opposed to applications which include skills that are abstract in nature or emphasize cognitive change (e.g., Socratic questioning). Similarly, when it comes to full therapy protocols, LLM applications for interventions that are highly structured, behavioral, and protocolized (e.g., CBT for insomnia [CBT-I] or exposure therapy for specific phobia) may be available sooner than applications delivering highly flexible or personalized interventions (for example^31^).

In theory, the final stage in the integration of LLMs into psychotherapy is fully autonomous delivery of psychotherapy which does not require human intervention or monitoring. However, it remains to be seen whether fully autonomous AI systems will reach a point at which they have been evaluated to be safe for deployment by the behavioral health community. Specific concerns include how well these systems are able to carry out case conceptualization on individuals with complex, highly comorbid symptom presentations, including accounting for current and past suicidality, substance use, safety concerns, medical comorbidities, and life circumstances and events (such as court dates and upcoming medical procedures). Similarly, it is unclear whether these systems will prove sufficiently adept at engaging patients over time^32^ or accounting for and addressing contextual nuances in treatment (e.g., using exposure to treat a patient experiencing PTSD-related fear of leaving the house, who also lives in a neighborhood with high rates of crime). Furthermore, several skills which may be viewed as central to clinical work currently fall outside the purview of LLM systems, such as interpreting nonverbal behavior (e.g., fidgeting, eye-rolling), appropriately challenging a patient, addressing alliance ruptures, and making decisions about termination. Technological advances, including the approaching advent of multimodal language models that integrate text, images, video, and audio, may eventually begin to fill these gaps.

Beyond technical limitations, it remains to be decided whether complete automation is an appropriate end goal for behavioral healthcare, due to safety, legal, philosophical, and ethical concerns^33^. While some evidence indicates that humans can develop a therapeutic alliance with chatbots^34^, the long-term viability of such alliance building, and whether or not it produces undesirable downstream effects (e.g., altering an individual’s existing relationships or social skills) remains to be seen. Others have documented potentially harmful behavior of LLM chatbots, such as narcissistic tendencies^35^ and expressed concerns about the potential for their undue influence on humans in addition to articulating societal risks associated with LLMs more generally^36,37^. The field will also need to grapple with questions of accountability and liability in the case of a fully autonomous clinical LLM application causing damage (e.g., identifying the responsible party in an incident of malpractice^38^). For these and other reasons, some have argued against the implementation of fully autonomous systems in behavioral healthcare and healthcare more broadly^39,40^. Taken together, these issues and concerns may suggest that in the short and medium term, assistive or collaborative AI applications will be more appropriate for the provision of behavioral healthcare.

## Applications of clinical LLMs

Given the vast nature of behavioral healthcare, there are seemingly endless applications of LLMs. Outlined below are some of the currently existing, imminently feasible, and potential long-term applications of clinical LLMs. Here we focus our discussion on applications directly related to the provision of, training in, and research on psychotherapy. As such, several important aspects of behavioral healthcare, such as initial symptom detection, psychological assessment and brief interventions (e.g., crisis counseling) are not explicitly discussed herein.

### Imminent applications

#### Automating clinical administration tasks

At the most basic level, LLMs have the potential to automate several time-consuming tasks associated with providing psychotherapy (Table 2, first row). In addition to using session transcripts to summarize the session for the provider, there is potential for such models to integrate within electronic health records to aid with clinical documentation and conducting chart reviews. Clinical LLMs could also produce a handout for the patient that provides a personalized overview of the session, skills learned and assigned homework or between-session material.

**Table 2:** Imminent possibilities for clinical LLMs

Task	Target Audience	Example Input to LLM	Example LLM Output
Aid in administrative tasks	Clinician	Psychotherapy session recording	“… Met with patient for cognitive behavioral therapy for depression. Reviewed homework; patient completed three thought records over the past week. Introduced ‘thinking biases’ worksheet; assisted patient in identifying patterns of problematic thinking applicable to her automatic negative thoughts from the past week…”
Offer feedback on therapy homework worksheets	Patient	Digital CBT worksheet; Patient writes, “I’ve always felt this way,” as evidence in support of the negative automatic thought: “I’m unlovable” on the worksheet	“Remember, ‘evidence’ means facts that support the belief. Sometimes it’s helpful to think about facts so strong they would stand up in a court of law. What is the evidence that you are unlovable?”
Produce adherence and competence ratings for elements of therapy	Researcher	Psychotherapy session recording	“*…Therapist helped patient identify negative automatic thoughts* Adherence rating (0-1): 1 Competence rating (0-6): 5…”
Identify trainee psychotherapist’s areas of success and areas for improvement	Psychotherapy trainee	Psychotherapy session recording	“…In the following exchange, the therapist successfully used Socratic questioning to ask open-ended, non-leading questions: *[Patient: I should have known that it wasn’t safe to get in that car. Therapist: Hm, help me understand… how could you have known that it wasn’t safe?]…”*
Suggest an improved therapeutic response, offer education about therapeutic exchanges	Peer counselor or lay mental health worker	Message-based exchange between patient and peer counselor; peer counselor has drafted a response: “You’ll be fine”	“This could be improved by offering validation of the client’s feelings. For instance, you might say, ‘it sounds like you’re going through a difficult time, and it’s understandable to feel overwhelmed.’ Would you like to rewrite before sending?”

#### Measuring treatment fidelity

A clinical LLM application could automate measurement of therapist fidelity to evidence-based practices (EBPs; Table 2, second row), which can include measuring *adherence* to the treatment as designed, *competence* in delivering a specific therapy skill, *treatment differentiation* (whether multiple treatments being compared actually differ from one another), and *treatment receipt* (patient comprehension of, engagement with, and adherence to the therapy content)^41,42^. Measuring fidelity is crucial to the development, testing, dissemination, and implementation of EBPs, yet can be resource intensive and difficult to do reliably. In the future, clinical LLMs could computationally derive adherence and competence ratings, aiding research efforts and reducing therapist drift^43^. Traditional machine-learning models are already being used to assess fidelity to specific modalities^44^ and other important constructs like counseling skills^45^ and alliance^46^. Given their improved ability to consider context, LLMs will likely increase the accuracy with which these constructs are assessed.

#### Offering feedback on therapy worksheets and homework

LLM applications could also be developed deliver real-time feedback and support on patients’ between-session homework assignments (Table 2, third row). For example, an LLM tailored to assist a patient to complete a CBT worksheet might provide clarification or aid in problem solving if the patient experiences difficulty (e.g., the patient was completing a thought log and having trouble differentiating between the thought and the emotion). This could help to “bridge the gap” between sessions and expedite patient skill development. Early evidence outside the AI realm^47^ points to increasing worksheet competence as a fruitful clinical target.

#### Automating aspects of supervision and training

LLMs could be used to provide feedback on psychotherapy or peer support sessions, especially for clinicians with less training and experience (i.e., peer counselors, lay health workers, psychotherapy trainees). For example, an LLM might be used to offer corrections and suggestions to the dialog of peer counselors (Table 2, fourth row). This application has parallels to “task sharing,” a method used in the global mental health field by which nonprofessionals provide mental health care with the oversight by specialist workers to expand access to mental health services^48^. Some of this work is already underway, for example, as described above, using LLMs to support peer counselors^7^.

LLMs could also support supervision for psychotherapists learning new treatments (Table 2, fifth row). Gold-standard methods of reviewing trainees’ work, like live observation or review of recorded sessions^49^, are time-consuming. LLMs could analyze entire therapy sessions and identify areas of improvement, offering a scalable approach for supervisors or consultants to review.

### Potential long-term applications

It is important to note that many of the potential applications listed below are theoretical and have yet to be developed, let alone thoroughly evaluated. Furthermore, we use the term “clinical LLM” in recognition of the fact that when and under what circumstances the work of an LLM could be called psychotherapy is evolving and depends on how psychotherapy is defined.

#### Fully autonomous clinical care

As previously described, the final stage of clinical LLM development could involve an LLM that can independently conduct comprehensive behavioral healthcare. This could involve all aspects related to traditional care including conducting assessment, presenting feedback, selecting an appropriate intervention and delivering a course of therapy to the patient. This course of treatment could be delivered in ways consistent with current models of psychotherapy wherein a patient engages with a “chatbot” weekly for a prescribed amount of time, or in more flexible or alternative formats. LLMs used in this manner would ideally be trained using standardized assessment approaches and manualized therapy protocols that have large bodies of evidence.

#### Decision aid for existing evidence-based practices

Even without full automation, clinical LLMs could be used as a tool to guide a provider on the best course of treatment for a given patient by optimizing the delivery of existing EBPs and therapeutic techniques. In practice, this may look like a LLM that can analyze transcripts from therapy sessions and offer a provider guidance on therapeutic skills, approaches or language, either in real time, or at the end of the therapy session. Furthermore, the LLM could integrate current evidence on the tailoring of specific EBPs to the condition being treated, and to demographic or cultural factors and comorbid conditions. Developing tailored clinical LLM “advisors” based on EBPs could both enhance fidelity to treatment and maximize the possibility of patients achieving clinical improvement in light of updated clinical evidence.

#### Development of new therapeutic techniques and EBPs

To this point, we have discussed how LLMs could be applied to current approaches to psychotherapy using extant evidence. However, LLMs and other computational methods could greatly enhance the detection and development of new therapeutic skills and EBPs. Historically, EBPs have traditionally been developed using human-derived insights and then evaluated through years of clinical trial research. While EBPs are effective, effect sizes for psychotherapy are typically small^50,51^ and significant proportions of patients do not respond^52^. There is a great need for more effective treatments, particularly for individuals with complex presentations or comorbid conditions. However, the traditional approach to developing and testing therapeutic interventions is slow, contributing to significant time lags in translational research^53^, and fails to deliver insights at the level of the individual.

Data-driven approaches hold the promise of revealing patterns that are not yet realized by clinicians, thus generating new approaches to psychotherapy; machine learning is already being used, for example, to predict behavioral health treatment outcomes^54^. With their ability to parse and summarize natural language, LLMs could add to existing data-driven approaches. For example, an LLM could be provided with a large historical dataset containing psychotherapy transcripts of different therapeutic orientations, outcome measures and sociodemographic information, and tasked with detecting therapeutic behaviors and techniques associated with objective outcomes (e.g., reduction in depressive symptoms). Using such a process might make it possible for an LLM to yield fine-grained insights about what makes existing therapeutic techniques work best (e.g., Which components of existing EBPs are the most potent? Are there therapist or patient characteristics that moderate the efficacy of intervention X? How does the ordering of interventions effect outcomes?) or even to isolate previously unidentified therapeutic techniques associated with improved clinical outcomes. By identifying what happens in therapy in such a fine-grained manner, LLMs could also play a role in revealing mechanisms of change, which is important for improving existing treatments and facilitating real-world implementation^55^.

However, to realize this possibility, and make sure that LLM-based advances can be integrated and vetted by the clinical community, it is necessary to steer away from the development of “black box,” LLM-identified interventions with low explainability (e.g., interpretability^56^). To guard against interventions with low interpretability, work to finetune LLMs to improve patient outcomes could include inspectable representations of the techniques employed by the LLM. Clinicians could examine these representations and situate them in the broader psychotherapy literature, which would involve comparing them to existing psychotherapy techniques and theories. Such an approach could speed up the identification of novel mechanisms while guarding against the identification of “novel” interventions which overlap with existing techniques or constructs (thus avoiding the jangle fallacy, the erroneous assumption that two constructs with different names are necessarily distinct^57^).

In the long run, by combining this information, it might even be possible for an LLM to “reverse-engineer” a new EBP, freed from the constraints of traditional therapeutic protocols and instead maximizing on the delivery of the constituent components shown to produce patient change (in a manner akin to modular approaches, wherein an individualized treatment plan is crafted for each patient by curating and sequencing treatment modules from an extensive menu of all available options based on the unique patient’s presentation^31^). Eventually, a self-learning clinical LLM might deliver a broad range of psychotherapeutic interventions while measuring patient outcomes and adapting its approach on the fly in response to changes in the patient (or lack thereof).

### Toward a precision medicine approach to psychotherapy

Current approaches to psychotherapy often are unable to provide guidance on the best approach to treatment when an individual has a complex presentation, which is often the rule rather than being the exception. For example, providers are likely to have greatly differing treatment plans for a patient with concurrent PTSD, substance use, chronic pain, and significant interpersonal difficulties. Models that use a data-driven approach (rather than a provider’s educated guess) to address an individual’s presenting concern alongside their comorbidities, sociodemographic factors, history, and responses to the current treatment, may ultimately offer the best chance at maximizing patient benefit. While there have been some advances in precision medicine approaches in behavioral healthcare^54,58^, these efforts are in their infancy and limited by sample sizes^59^.

The potential applications of clinical LLMs we have outlined above may come together to facilitate a personalized approach to behavioral healthcare, analogous to that of precision medicine. Through optimizing existing EBPs, identifying new therapeutic approaches, and better understanding mechanisms of change, LLMs (and their future descendants) may provide behavioral healthcare with an enhanced ability to identify what works best for whom and under what circumstances.

## Recommendations for responsible development and evaluation of clinical LLMs

### Focus first on evidence-based practices

In the immediate future, clinical LLM applications will have the greatest chance of creating meaningful clinical impact if developed based on EBPs or a “common elements” approach (i.e., evidence-based procedures shared across treatments)^60^. Evidence-based treatments and techniques have been identified for specific psychopathologies (e.g., major depressive disorder, posttraumatic stress disorder), stressors (e.g., bereavement, job loss, divorce), and populations (e.g., LGBTQ individuals, older adults)^55,61,62^. Without an initial focus on EBPs, clinical LLM applications may fail to reflect current knowledge and may even produce harm^63^. Only once LLMs have been fully trained on EBPs can the field start to consider using LLMs in a data-driven manner, such as those outlined in the previous section on potential long-term applications.

### Focus next on improvement (engagement is not enough)

Others have highlighted the importance of promoting engagement with digital mental health applications^15^, which is important for achieving an adequate “dose” of the therapeutic intervention. LLM applications hold the promise of improving engagement and retention through their ability to respond to free text, extract key concepts, and address patients’ unique context and concerns during interventions in a timely manner. However, engagement alone is not an appropriate outcome on which to train an LLM, because engagement is not expected to be sufficient for producing change. A focus on such metrics for clinical LLMs will risk losing sight of the primary goals, clinical improvement (e.g., reductions in symptoms or impairment, increases in well-being and functioning) and prevention of risks and adverse events. It will behoove the field to be wary of attempts to optimize clinical LLMs on outcomes that have an explicit relationship with a company’s profit (e.g., length of time using the application). An LLM that optimizes only for engagement (akin to YouTube recommendations) could have high rates of user retention without employing meaningful clinical interventions to reduce suffering and improve quality of life. Previous research has suggested that this may be happening with non-LLM digital mental health interventions. For instance, exposure is a technique with strong support for treating anxiety, yet it is rarely included in popular smartphone applications for anxiety^64^, perhaps because developers fear that the technique will not appeal to users, or have concerns about how exposures going poorly or increasing anxiety in the short term, which may prompt concerns about legal exposure.

### Commit to rigorous yet commonsense evaluation

An evaluation approach for clinical LLMs that hierarchically prioritizes risk and safety, followed by feasibility, acceptability, and effectiveness, would be in line with existing recommendations for the evaluation of digital mental health smartphone apps^65^. The first level of evaluation could involve a demonstration that a clinical LLM produces no harm or very minimal harm that is outweighed by its benefits, similar to FDA phase I drug tests. Key risk and safety related constructs include measures of suicidality, non-suicidal self harm, and risk of harm to others.

Next, rigorous examinations of clinical LLM applications will be needed to provide empirical evidence of their utility, using head-to-head comparisons with standard treatments. Key constructs to be assessed in these empirical tests are feasibility and acceptability to the patient and the therapist as well as treatment outcomes (e.g., symptoms, impairment, clinical status, rates of relapse). Other relevant considerations include patients’ user experience with the application, measures of therapist efficiency and burnout, and cost.

Lastly, we note that given that possible benefits of clinical LLMs (including expanding access to care), it will be important for the field to adopt a commonsense approach to evaluation. While rigorous evaluation is important, the comparison conditions on which these evaluations are based should reflect real-world risk and efficacy rates, and perhaps employ a graded hierarchy with which to classify risk and error (i.e., missing a mention of suicidality is unacceptable, but getting a patient’s partner’s name wrong is nonideal but tolerable), rather than holding clinical LLM applications to a standard of perfection which humans do not achieve. Furthermore, developers will need to strike the appropriate balance of prioritizing constructs in a manner expected to be most clinically beneficial, for example, if exposure therapy is indicated for the patient, but the patient does not find this approach acceptable, the clinical LLM could recommend the intervention prioritizing effectiveness before offering second-line interventions which may be more acceptable.

### Involve interdisciplinary collaboration

Interdisciplinary collaboration between clinical scientists, engineers, and technologists will be crucial in the development of clinical LLMs. While it is plausible that engineers and technologists could use available therapeutic manuals to develop clinical LLMs without the expertise of a behavioral health expert, this is ill-advised. Manuals are only a first step towards learning a specific intervention, as they do not provide guidance on how the intervention can be applied to specific individuals or presentations, or how to handle specific issues or concerns that may arise through the course of treatment.

Clinicians and clinician-scientists have expertise that bears on these issues, as well as many other aspects of the clinical LLM development process. Their involvement could include a) testing new applications to identify limitations and risks and optimize their integration into clinical practice, b) improving the ability of applications to adequately address the complexity of psychological phenomena, c) ensuring that applications are developed and implemented in an ethical manner, and d) testing and ensuring that applications don’t have iatrogenic effects, such as reinforcing behaviors that perpetuate psychopathology or distress.

Behavioral health experts could also provide guidance on how best to finetune or tailor models, including addressing the question of whether and how real patient data should be used for these purposes. For example, most proximately, behavioral health experts might assist in *prompt engineering*, or the designing and testing of a series of prompts which provide the LLM framing and context for delivering a specific type of treatment or clinical skill (e.g., “Use cognitive restructuring to help the patient evaluate and reappraise negative thoughts in depression”), or a desired clinical task, such as evaluating therapy sessions for fidelity (e.g., “Analyze this psychotherapy transcript and select sections in which the therapist demonstrated the particularly skillful use of CBT skills, and sections in which the therapist’s delivery of CBT skills could be improved”). Similarly, in *few-shot learning*, behavioral health experts could be involved in crafting example exchanges which are added to prompts. For example, treatment modality experts might generate examples of clinical skills (e.g., high-quality examples of using cognitive restructuring to address depression) or of a clinical task (e.g., examples of both high- and low-quality delivery of CBT skills). For *fine-tuning*, in which a large, labeled dataset is used to train the LLM, and *reinforcement learning from human feedback* (RLHF), in which a human-labeled dataset is used to train a smaller model which is then used for LLM “self-training,” behavioral health experts could build and curate (and ensure informed patient consent for use of) appropriate datasets (e.g., a dataset containing psychotherapy transcripts rated for fidelity to an evidence-based psychotherapy). The expertise that behavioral health experts could draw on to generate instructive examples and curate high-quality datasets holds particular value in light of recent evidence that *quality* of data trumps *quantity* of data for training well-performing models^66^.

In the service of facilitating interdisciplinary collaboration, it would benefit clinical scientists to seek out a working knowledge about LLMs, while it would benefit technologists to develop a working knowledge of therapy in general and EBPs in particular. Dedicated venues that bring together behavioral health experts and clinical psychologists for interdisciplinary collaboration and communication will aid in these efforts. Historically, venues of this type have included psychology-focused workshops at NLP conferences (e.g., the Workshop on Computational Linguistics and Clinical Psychology [CLPsych], held at the Annual Conference of the North American Chapter of the Association for Computational Linguistics [NAACL]) and technology-focused conferences or workgroups hosted by psychological organizations (e.g., APA’s Technology, Mind & Society conference; Association for Behavioral and Cognitive Therapies’ [ABCT] Technology and Behavior Change special interest group). This work has also been done at nonprofits centered on technological tools for mental health (e.g., the Society for Digital Mental Health). Beyond these venues, it may be fruitful to develop a gathering that brings together technologists, clinical scientists, and industry partners with a dedicated focus on AI/LLMs, which could routinely publish on its efforts, akin to the efforts of the World Health Organization’s Infodemic Management Conference, which has employed this approach to address misinformation^67^. Finally, given the numerous applications of AI to behavioral health, it is conceivable that a new “computational behavioral health” subfield could emerge, offering specialized training that would bridge the gap between these two domains.

### Focus on trust and usability for clinicians and patients

It is important to engage therapists, policymakers, end-users, and experts in human-computer interactions to understand and improve levels of trust that will be necessary for successful and effective implementation. With respect to applications of AI to augment supervision and support for psychotherapy, therapists have expressed concern about privacy, the ability to detect subtle non-verbal cues and cultural responsiveness, and the impact on therapist confidence, but they also see benefits for training and professional growth^68^. Other research suggests that while therapists believe AI can increase access to care, allow individuals to disclose embarrassing information more comfortably, continuously refine therapeutic techniques^69^, they have concerns about privacy and the formation of a strong therapeutic bond with machine-based therapeutic interventions^70^. Involvement of individuals who will be referring their patients and using LLMs in their own practice will be essential to developing solutions they can trust and implement, and to make sure these solutions have the features that support trust and usability (simple interfaces, accurate summaries of AI-patient interactions, etc.).

Regarding how much patients will trust the AI systems, following the stages we outlined in Fig. 3, initial AI-patient interactions will continue to be supervised by clinicians, and the therapeutic bond between the clinician and the patient will continue to be the primary relationship. During this stage, it is important that clinicians talk to the patients about their experience with the LLMs, and that the field as a whole begins to accumulate an understanding and data on how acceptable interfacing with LLMs is for what kind of patient for what kind of clinical use case, in how clinicians can scaffold the patient-LLM relationship. This data will be critical for developing collaborative LLM applications that have more autonomy, and for ensuring that the transition from assistive to collaborative stage applications is not associated with large unforeseen risk. For example, in the case of CBT for insomnia, once an assistive AI system has been iterated on to reliably collect information about patients’ sleep patterns, it is more conceivable that it could be evolved into a collaborative AI system that does a comprehensive insomnia assessment (i.e., it also collects and interprets data on patients’ clinically significant distress, impairment of functioning, and ruling out of sleep-wake disorders, like narcolepsy)^71^.

### Design criteria for effective clinical LLMs

Below, we propose an initial set of desirable design qualities for clinical LLMs.

#### Detect risk of harm

- Accurate risk detection and mandated reporting are crucial aspects that clinical LLMs must prioritize, particularly in the identification of suicidal/homicidal ideation, child/elder abuse, and intimate partner violence. Algorithms for detecting risks are under development^4^. One threat to risk detection is that current LLMs have limited context windows, meaning they only “remember” a limited amount of user input. Functionally, this means a clinical LLM application could “forget” crucial details about a patient, which could impact safety (e.g., an application “forgetting” that the patient owns firearms would threaten its ability to properly assess and intervene around suicide risk). However, context windows have been rapidly expanding with each subsequent model release, so this issue may not be a problem for long. In addition, it is already possible to augment the memory of LLMs with “vector databases,” which would have the added benefit of retaining inspectable learnings and summaries across clinical encounters^72^.

In the future, and especially given much larger context windows, clinical LLMs could prompt clinicians with ethical guidelines, legal requirements (e.g., the Tarasoff rule, which requires clinicians to warn intended victims when a patient presents a serious threat of violence), or evidence-based methods for decreasing risk (e.g., safety planning^73^), or even provide interventions targeting risk directly to patients. This type of risk monitoring and intervention could be particularly useful in supplementing existing healthcare systems during gaps in clinician coverage like nights and weekends^4^.

b) Be “Healthy.” There is growing concern that AI chat systems can demonstrate undesirable behaviors, including expressions akin to depression or narcissism^35,74^. Such poorly understood, undesirable behaviors risk harming already vulnerable patients or interfering with their ability to benefit from treatment. Clinical LLM applications will need training, monitoring, auditing, and guardrails to prevent the expression of undesirable behaviors and maintain healthy interactions with users. These efforts will need to be continually evaluated and updated to prevent or address the emergence of new undesirable or clinically contraindicated behavior.

#### Aid in psychodiagnostic assessment

Clinical LLMs ought to integrate psychodiagnostic assessment and diagnosis, facilitating intervention selection and outcome monitoring^75^. Recent developments show promise for LLMs in the assessment realm^76^. Down the line, LLMs could be used for diagnostic interviewing (e.g., Structured Clinical Interview for the *DSM-5*^77^) using chatbots or voice interfaces. Prioritizing assessment enhances diagnostic accuracy and ensures appropriate intervention, reducing the risk of harmful interventions^63^.

#### Be responsive and flexible

Given the frequency with which ambivalence and poor patient engagement arise in clinical encounters, clinical LLMs which use evidence-based and patient-centered methods for handling these issues (e.g., motivational enhancement techniques, shared decision making), and have options for second-line interventions for patients not interested in gold-standard treatments, will have the best chance of success.

#### Stop when not helping or confident

Psychologists are ethically obligated to cease treatment and offer appropriate referrals to the patient if the current course of treatment has not helped or likely will not help. Clinical LLMs can abide by this ethical standard by drawing on integrated assessment (discussed above) to assess the appropriateness of the given intervention and detect cases that need more specialized or intensive intervention.

#### Be fair, inclusive, and free from bias

As has been written about extensively, LLMs may perpetuate bias, including racism, sexism, and homophobia, given that they are trained on existing text^36^. These biases can contribute to both error disparities – where models are less accurate for particular groups – or outcome disparities – where models tend to over-capture demographic information^78^ – which would in turn contribute to the disparities in mental health status and care already experienced by minoritized groups^79^. The integration of bias countermeasures into clinical LLM applications could serve to prevent this^78,80^.

#### Be empathetic–to an extent

Clinical LLMs will likely need to demonstrate empathy and build the therapeutic alliance in order to engage patients. Other skills used by therapists include humor, irreverence, and gentle methods of challenging the patient. Incorporating these into clinical LLMs might be beneficial, as appropriate human likeness may facilitate engagement and interaction with AI^81^. However, this needs to be balanced against associated risks, mentioned above, of incorporating human likeness in systems^36^. Whether and how much human likeness is necessary for a psychological intervention remains a question for future empirical work.

#### Be transparent about being AIs

Mental illness and mental health care is already stigmatized, and the application of LLMs without transparent consent can erode patient/consumer trust, which reduces trust in the behavioral health profession more generally. Some mental health startups have already faced criticism for employing generative AI in applications without disclosing this information to the end user^2^. As laid out in the White House Blueprint for an AI Bill of Rights, AI applications should be explicitly (and perhaps repeatedly/consistently) labeled as such to allow patients and consumers to “know that an automated system is being used and understand how and why it contributes to outcomes that impact them”^82^.

## Discussion

### Unintended consequences may change the clinical profession

The development of clinical LLM applications could lead to unintended consequences, such as changes to the structure of and compensation for mental health services. AI may permit increased staffing by non-professionals or paraprofessionals, causing professional clinicians to supervise large numbers of non-professionals or even semi-autonomous LLM systems. This could reduce clinicians’ direct patient contact and perhaps increase their exposure to challenging or complicated cases not suitable for the LLM, which may lead to burnout and make clinical jobs less attractive. To address this, research could determine the appropriate number of cases for a clinician to oversee safely and guidelines could be published to disseminate these findings. The 24-hour availability of LLM-based intervention may also change consumer expectations of psychotherapy in a way that is at odds with many of the norms of psychotherapy practice (e.g., waiting for a session to discuss stressors, limited or emergency-only contact between sessions).

### LLMs could pave the way for a next generation of clinical science

Beyond the imminent applications described in this paper, it is worth considering how the long-term applications of clinical LLMs might also facilitate significant advances in clinical care and clinical science.

#### Clinical practice

In terms of their effects on therapeutic interventions themselves, clinical LLMs might promote advances in the field by allowing for the pooling of data on what works with the most difficult cases, perhaps through the use of practice research networks^83^. At the level of health systems, they could expedite the implementation and translation of research findings into clinical practice by suggesting therapeutic strategies to psychotherapists, for instance, promoting strategies that enhance inhibitory learning during exposure therapy^84^. Lastly, clinical LLMs could increase access to care if LLM-based psychotherapy chatbots are offered as low intensity, low-cost options in stepped-care models, similar to the existing provision of computerized CBT and guided self-help^85^.

As the utilization of clinical LLMs expands, there may be a shift towards psychologists and other behavioral health experts operating at the top of their degree. Presently, a significant amount of clinician time is consumed by administrative tasks, chart review, and documentation. The shifting of responsibilities afforded by the automation of certain aspects of psychotherapy by clinical LLMs could allow clinicians to pursue leadership roles, contribute to the development, evaluation, and implementation of LLM-based care, or lead policy efforts, or simply to devote more time to direct patient care.

#### Clinical science

By facilitating supervision, consultation, and fidelity measurement, LLMs could expedite psychotherapist training and increase the capacity of study supervisors, thus making psychotherapy research less expensive and more efficient.

In a world in which fully autonomous LLM applications screen and assess patients, deliver high-fidelity, protocolized psychotherapy, and collect outcome measurements, psychotherapy clinical trials would be limited largely by the number of willing participants eligible for the study, rather than by the resources required to screen, assess, treat, and follow these participants. This could open the door to unprecedentedly large-N clinical trials. This would allow for well-powered, sophisticated dismantling studies to support the search for mechanisms of change in psychotherapy, which are currently only possible using individual participant level meta-analysis (for example, see ref. 86). Ultimately, such insights into causal mechanisms of change in psychotherapy could help to refine these treatments and potentially improve their efficacy.

Finally, the emergence of LLM treatment modalities will challenge (or confirm) fundamental assumptions about psychotherapy. Does therapeutic (human) alliance account for a majority of the variance in patient change? To what extent can an alliance be formed with a technological agent? Is lasting and meaningful therapeutic change only possible through working with a human therapist? LLMs hold the promise of empirical answers to these questions.

In summary, large language models hold promise for supporting, augmenting, or even in some cases replacing human-led psychotherapy, which may improve the quality, accessibility, consistency, and scalability of therapeutic interventions and clinical science research. However, LLMs are advancing quickly and will soon be deployed in the clinical domain, with little oversight or understanding of harms that they may produce. While cautious optimism about clinical LLM applications is warranted, it is also crucial for psychologists to approach the integration of LLMs into psychotherapy with caution and to educate the public about the potential risks and limitations of using these technologies for therapeutic purposes. Furthermore, clinical psychologists ought to actively engage with the technologists building these solutions. As the field of AI continues to evolve, it is essential that researchers and clinicians closely monitor the use of LLMs in psychotherapy and advocate for responsible and ethical use to protect the wellbeing of patients.

## Data Availability

Data sharing not applicable to this article as no datasets were generated or analyzed during the current study.
